# Quantitative evaluation of carotid atherosclerotic vulnerable plaques using in vivo T1 mapping cardiovascular magnetic resonaonce: validation by histology

**DOI:** 10.1186/s12968-020-00624-0

**Published:** 2020-05-21

**Authors:** Huiyu Qiao, Dongye Li, Jingli Cao, Haikun Qi, Yongjun Han, Hualu Han, Huimin Xu, Tao Wang, Shuo Chen, Huijun Chen, Yajie Wang, Xihai Zhao

**Affiliations:** 1 Center for Biomedical Imaging Research, Department of Biomedical Engineering, Tsinghua University School of Medicine, Haidian District, Beijing, 100084 China; 2 Department of Radiology, Sun Yat-sen Memorial hospital, Sun Yat-sen University, Guangzhou, China; 3 China National Clinical Research Center for Neurological Diseases, Beijing Tiantan Hospital, Capital Medical University, Beijing, China; 4 School of Biomedical Engineering and Imaging Sciences, King’s College London, London, UK; 5 Department of Radiology, Peking University Third Hospital, Beijing, China; 6 Department of Neurosurgery, Peking University Third Hospital, Beijing, China; 7 Department of Clinical Laboratory, Beijing Ditan Hospital, Capital Medical University, Beijing, China

**Keywords:** Carotid artery, Atherosclerosis, Intraplaque hemorrhage, T1 mapping, Magnetic resonance imaging

## Abstract

**Background:**

It has been proved that multi-contrast cardiovascular magnetic resonance (CMR) vessel wall imaging could be used to characterize carotid vulnerable plaque components according to the signal intensity on different contrast images. The signal intensity of plaque components is mainly dependent on the values of T1 and T2 relaxation. T1 mapping recently showed a potential in identifying plaque components but it is not well validated by histology. This study aimed to validate the usefulness of in vivo T1 mapping in assessing carotid vulnerable plaque components by histology.

**Methods:**

Thirty-four subjects (mean age, 64.0 ± 8.9 years; 26 males) with carotid plaques referred to carotid endarterectomy were prospectively enrolled and underwent 3 T CMR imaging from May 2017 to October 2017. The T1 values of intraplaque hemorrhage (IPH), necrotic core (NC) and loose matrix (LM) which were identified on multi-contrast vessel wall images or histology were measured on in-vivo T1 mapping. The IPHs were divided into two types based on the proportion of the area of fresh hemorrhage on histology. The T1 values of different plaque components were compared using Mann-Whitney U test and the agreement between T1 mapping and histology in identifying and quantifying IPH was analyzed with Cohen’s Kappa and intraclass correlation coefficient (ICC).

**Results:**

Of 34 subjects, 19 had histological specimens matched with CMR imaging. The mean T1 values of IPH (651 ± 253 ms), NC (1161 ± 182 ms) and LM (1447 ± 310 ms) identified by histology were significantly different. The T1 values of Type 1 IPH were significantly shorter than that of Type 2 IPH (456 ± 193 ms vs. 775 ± 205 ms, *p* < 0.001). Moderate to excellent agreement was found in identification (kappa = 0.51, *p* < 0.001), classification (kappa = 0.40, *p* = 0.028) and segmentation (ICC = 0.816, 95% CI 0.679–0.894) of IPHs between T1 mapping and histology.

**Conclusions:**

The T1 values of carotid plaque components, particularly for intraplaque hemorrhage, are differentiable, and the stage of intraplaque hemorrhage can be classified according to T1 values, suggesting the potential capability of assessment of vulnerable plaque components by T1 mapping.

## Background

It is well evidenced that cerebrovascular vulnerable atherosclerotic plaque is the key etiology of ischemic stroke [1]. Histologically, intraplaque hemorrhage (IPH) and large necrotic core (NC) have been considered as the key features of vulnerable plaques [2]. In addition, investigators found that stage of IPH was associated with cardiovascular events [3]. Therefore, it is important to accurately characterize vulnerable plaque components.

Multi-contrast cardiovascular magnetic resonance (CMR) vessel wall imaging, including time-of-flight (TOF), T1-weighted, and T2-weighted sequences, has become an ideal non-invasive technique to assess carotid plaque components [4]. In multi-contrast CMR imaging techniques, each sequence plays a specific role in the identification of specific plaque component. Pre- and post-contrast T1-weighted imaging has been largely used to evaluate NC with good agreement with histology [5]. The sequence of magnetization prepared rapid gradient echo acquisition (MP-RAGE) has been demonstrated to be the best technique in characterizing IPH among three different T1-weighted sequences [6]. It is evidenced that the stage of carotid artery IPH can be distinguished by combining TOF, T1-weighted, and T2-weighted images [7].

Since plaque components are determined according to their signal intensity on different contrast images and CMR signal is mainly dependent on the values of T1 and T2 relaxation, it is possible to distinguish plaque components by quantitative imaging. Recently, quantitative CMR imaging techniques, such as T1 mapping, have been proposed for plaque characterization [8–11]. Although investigators utilized ex-vivo T1 mapping to assess intracranial plaques [8], few studies investigated the in-vivo T1 values of carotid plaque components. Ota and colleagues measured the in-vivo longitudinal relaxation rates (R1 = 1/T1) of carotid homogenous plaque components which could not be applied to characterization of those plaques with inhomogeneous and complicated components [9]. Subsequently, Qi et al. developed a 3D SNAP with golden angle radial k-space sampling (GOAL-SNAP) sequence for measuring T1 values of vessel wall and this technique showed a potential in identifying IPH and monitoring its progression [10]. However, the capability of GOAL-SNAP sequence in characterizing vulnerable plaques has not been validated by histology.

Here, we hypothesized that different plaque components have different T1 values and the T1 values of vessel wall can be used to identify vulnerable plaque features. This study sought to validate the usefulness of in vivo T1 mapping in assessing carotid vulnerable plaque compositional features by histology.

## Methods

### Study sample

Thirty-four subjects (mean age, 64.0 ± 8.9 years; 26 males) with 50–70% symptomatic stenosis or ≥ 70% stenosis in carotid artery referred to carotid endarterectomy (CEA) were prospectively and consecutively enrolled from Peking University Third Hospital during May 2017 to October 2017. All subjects underwent CMR imaging for carotid arteries within 1 week before CEA surgery. The exclusion criteria are: 1) stenting therapy; and 2) contraindications to CMR examination. Clinical information including age, gender, height, weight, blood pressure, hyperlipidemia, hypertension, diabetes, and history of cardiovascular disease were collected from medical records. The study protocol was approved by local institutional review board and written informed consent form was obtained from each subject.

### Carotid CMR imaging

Carotid arteries of all subjects were imaged on 3 T CMR scanner (Achieva TX, Philips Healthcare, Best, The Netherlands) with a dedicated 8-channel carotid coil. CMR vessel wall imaging including T1-quadruple inversion recovery (QIR), T2-multislice double inversion recovery (MDIR), 3D TOF and MP-RAGE sequences and 3D T1 mapping sequence (GOAL-SNAP) [10] was performed centered to the bifurcation of carotid artery which was the target of CEA surgery. The imaging parameters are presented in Table 1.

**Table 1:** CMR vessel wall imaging and GOAL-SNAP imaging parameters

	Multi-contrast CMR imaging				T1 Mapping
	T1-QIR	T2-MDIR	3D TOF	MP-RAGE	GOAL-SNAP
Acquisition	TSE	TSE	FFE	TFE	TFE
TR/TE (ms)	800/10	4800/50	20/5.5	13.2/3.2	11.3/4.7
Turbo factor	10	12	–	55	175
Flip angle (deg)	90	90	20	15	8
FOV (mm^3^)	160 × 160 × 32		160 × 160 × 48		100 × 100 × 100
Resolution (mm^3^)^a^	0.6 × 0.6 × 2		0.6 × 0.6 × 2		0.8 × 0.8 × 0.8
Orientation	Axial	Axial	Axial	Axial	Axial
NSA	1	1	1	2	1
Scan time (min)	6	4	2	4	5

*TSE* turbo spin echo, *FFE* fast field echo, *TFE* turbo field echo, *TR* repetition time, *TE* echo time, *FOV* field of view, *NSA* number of signal averaged, *GOAL-SNAP* SNAP with golden angle radial k-space sampling

^a^Acquisition resolution

### CMR image analysis

The images of GOAL-SNAP were off-line reconstructed to inversion recovery (IR) image series using sliding window and the k-space weighted image contrast (KWIC) methods for T1 fitting [12]. To compare with multi-contrast CMR images, the reconstructed 3D GOAL-SNAP images were reformatted to 2 mm axial slices with the same off-center distance and angulation to T1-QIR images using a Philips MR workstation (Extended MR Workspace 2.6.3.1, Philips Medical System). This process can ensure that images with the same number of slices from these two sequences had same geometric position. The reformatted IR images series from GOAL-SNAP and T1-QIR images were registered for each subject in “CASCADE” software (UW, Seattle, Washington, USA) [13]. The registration between GOAL-SNAP and T1-QIR images was performed by combining the auto registration in software and manual adjustment. The multi-contrast CMR images were reviewed by two radiologists with > 5 years’ experience in CMR using custom-designed software of “CASCADE” with consensus. The CMR image quality was assessed with 4-point scale: 1 = poor; 2 = marginal; 3 = good; and 4 = excellent [14]. Slices with image quality < 2 were excluded. The boundaries of lumen and outer wall were outlined on each axial slice. The carotid plaque components, including loose matrix (LM), IPH and NC, were identified and their areas were measured on the multi-contrast CMR vessel wall images using the published criteria [4]. The T1 fitting was conducted using the reformatted and registered GOAL-SNAP IR images in the wall regions for each artery. According to the label masks which were obtained from multi-contrast images and the fitted T1 maps, the T1 values of different plaque components were recorded. The reconstruction and T1 fitting of GOAL-SNAP images were performed using MATLAB 2014 (MathWorks, Inc. Natick, Massachusetts, USA).

### Process and analysis of histological specimen

**Fig. 1:**
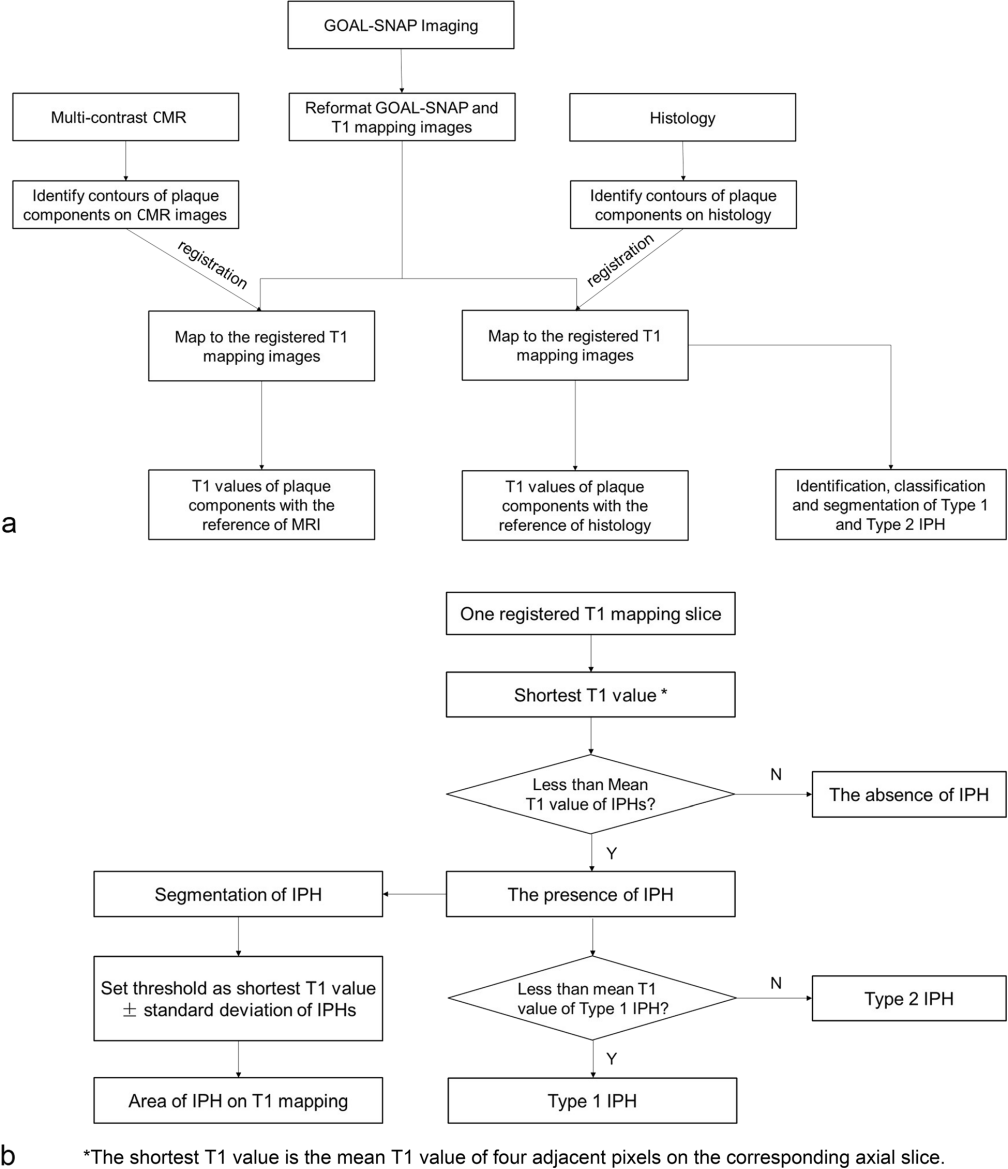
The flowchart of CMR image analysis, histologic sample analysis (**a**) and intraplaque hemorrhage assessments (**b**). The shortest T1 value was defined as the mean T1 value of four adjacent pixels with the lowest T1 value to avoid measuring noise. The mean T1 value of intraplaque hemorrhage (IPH), mean T1 values of Type 1 IPH and the standard deviation of IPH were obtained from histological analysis

After CEA, the excised plaques were fixed in 10% neutral buffered formalin within 4 h, decalcified in 10% formic acid, and embedded a bloc in paraffin. The specimen was sectioned with thickness of 10 μm every 0.5 mm and stained with hematoxylin-eosin (H&E). The histologic sections were matched with the CMR images by experienced pathologist and radiologist according to the position and the shape of lumen, wall, and plaques with the landmarks of bifurcation. The LM, IPH and NC were identified and quantified on each matched histologic section by two experienced pathologists with > 5 years’ experience using ImageJ software and published criteria with consensus [15, 16]. The IPHs were classified into two types according to the proportion of the area of fresh hemorrhage occupying the total area of IPH on each section: Type 1, ≥50%; Type 2, < 50%. The proportion of the area of fresh hemorrhage occupying the total area of IPH was also recorded on each section. All the histological marks of LM, IPH, NC were mapped onto the registered T1 mapping images and the corresponding T1 values were recorded on each registered slice. Sixteen histological specimens from 16 patients were randomly selected for reproducibility study. The same two pathologists identified plaque components with consensus blinded to the first round of review with one-year time interval to minimize the memory bias. The corresponding marks were mapped onto the registered T1 mapping images and the T1 values of LM, NC and IPH on these 16 slices were recorded. The classification of IPH was also conducted in the reproducibility study and compared with previous evaluation. The flowchart of analysis of CMR images and histologic sections is shown in Fig. 1a.

### Assessment of IPHs, LM and NC with T1 mapping

The mean T1 values of two types of IPHs which were obtained from above histological analysis were used as cut-off values to identify and classify different types of IPH. For each matched slice, the shortest T1 value was defined as the mean T1 value of four adjacent pixels with the lowest T1 value to avoid measuring noise. If the shortest T1 value of the axial slice was shorter than the mean T1 value of both types of IPHs, the presence of IPH will be identified on the corresponding T1 mapping slice. Among the slices with IPHs, the slices in which the shortest T1 value was shorter than the mean T1 value of Type 1 IPHs were classified into Type 1 IPH. In contrast, the slices in which the shortest T1 value was between the mean T1 value of Type 1 IPHs and whole IPHs were classified into Type 2 IPH. The segmentation of IPHs was performed on these slices with IPHs. The threshold-based segmentation of IPHs was also performed. The threshold for the segmentation was set as the sum of the shortest T1 value and 1 standard deviation of T1 values of IPHs (Fig. 1b).

The identification of LM or NC was based on the hypothesis that the T1 value of the center of LM or NC should be around the mean T1 value of the whole LM or NC. The mean T1 value and standard deviation of T1 value of LM or NC were obtained from previous histological analysis. The cut-off T1 value for the identification of LM or NC was set as mean ± one tenth of standard deviation of T1 value of LM or NC. One tenth of standard deviation of T1 value of LM or NC was set as a bound to ensure an appropriate size of the connected regions. If there is a region whose mean T1 value of at least three adjacent pixels was within the threshold range of LM or NC, the corresponding plaque component will be identified.

### Statistical analysis

The continuous variables were described as mean ± standard deviation and categorical variables were described as percentage. The T1 values of LM, IPHs and NC identified by multi-contrast images and histology were calculated and compared using one-way ANOVA and Mann-Whitney U tests, respectively. The agreement of T1 values between two different rounds of review on histological specimen was analyzed using intraclass correlation coefficient (ICC). The accuracy, sensitivity and specificity of above threshold of T1 value in identifying plaque components were calculated by comparing with the gold standard of histological results using diagnostic test. The agreements in identification of the presence and types of IPHs between T1 mapping and histology were analyzed using Cohen’s Kappa test. The correlation between T1 values and the proportion of the area of fresh hemorrhage occupying the total area of IPH on each section was evaluated using Pearson correlation analysis. The comparison and agreement of the area of IPHs between T1 mapping and histology were performed and determined by using paired *t* test and ICC analysis, respectively. The area of IPHs measured by histology was adjusted according to the potential shrinkage (7.8%) [17]. All statistical analyses were performed using SPSS (v. 16.0 Statistical Package for the Social Sciences, International Business Machines, Inc., Armonk, New York, USA). *P* values < 0.05 were considered statistically significant.

## Results

Of 34 recruited patients, 15 (44.1%) had hypertension, 3 (8.8%) had diabetes, 2 (5.9%) had hyperlipidemia, and 22 (64.7%) had a history of cardiovascular disease. Of 34 subjects, 1 failed to receive CEA surgery due to perioperative cardiac event and 4 had no intact specimens because of operative procedure. In total, 29 qualified specimens were collected for analysis. The clinical information is detailed in Table 2.

**Table 2:** Clinical information of study population (*n* = 34)

	Mean ± SD or n (%)	Range
Gender, male	26 (76.5)	
Age, years	64.0 ± 8.9	47–81
Body mass index, kg/m^2^	24.7 ± 3.5	18.7–30.8
Diabetes	3 (8.8)	
Hypertension	15 (44.1)	
Systolic blood pressure, mmHg	141.5 ± 19.1	105–201
Diastolic blood pressure, mmHg	74.3 ± 10.6	53–93
Hyperlipidemia	2 (5.9)	
History of cardiovascular disease	22 (64.7)	

### T1 values of plaque components based-on masks of multi-contrast CMR images

**Fig. 2:**
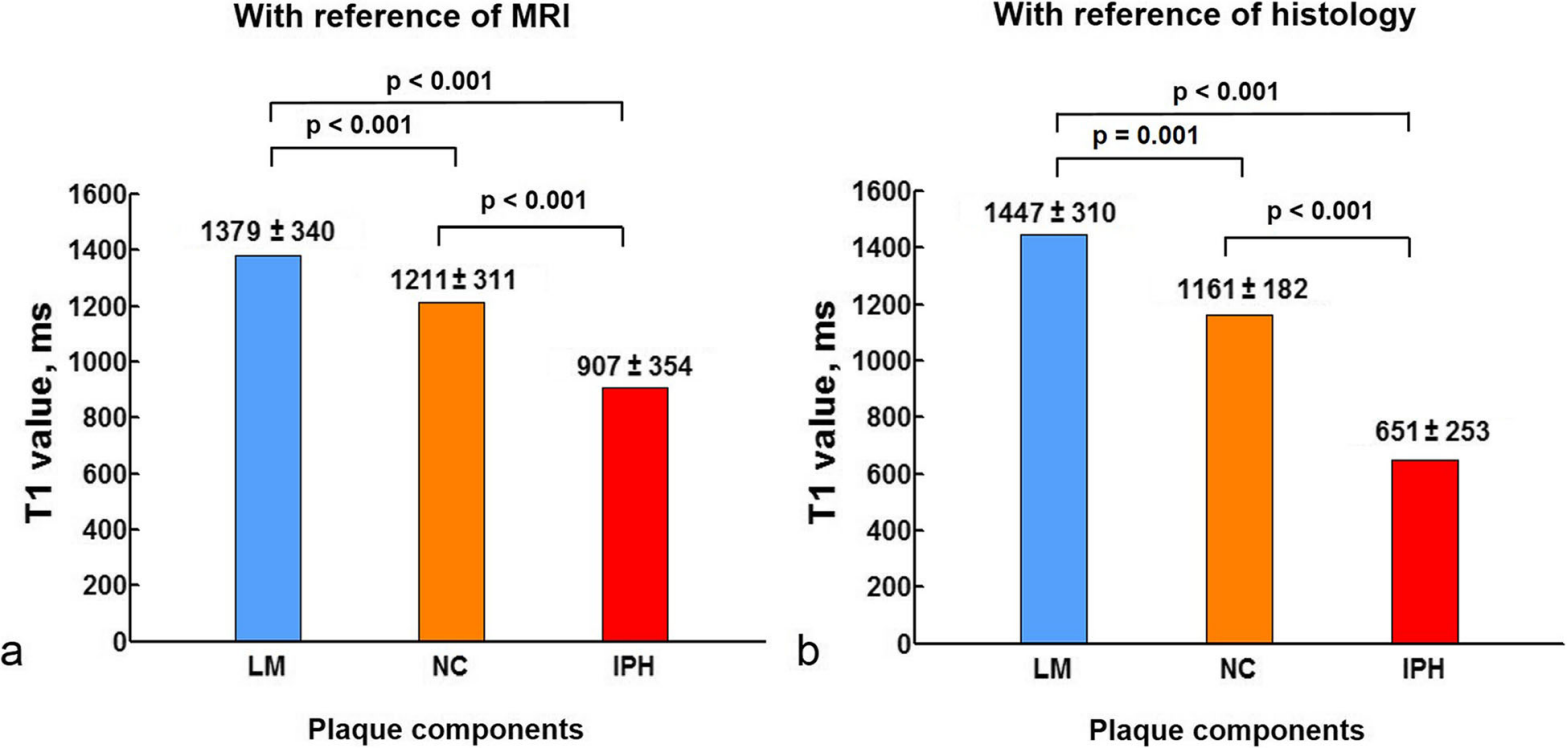
The plaque components' T1 values calculated with reference of multi-contrast CMR imaging (**a**) and histology (**b**). The carotid plaque components included loose matrix (LM), necrotic core (NC) and intraplaque hemorrhage (IPH). IPH appeared the significantly lowest T1 value

**Fig. 3:**
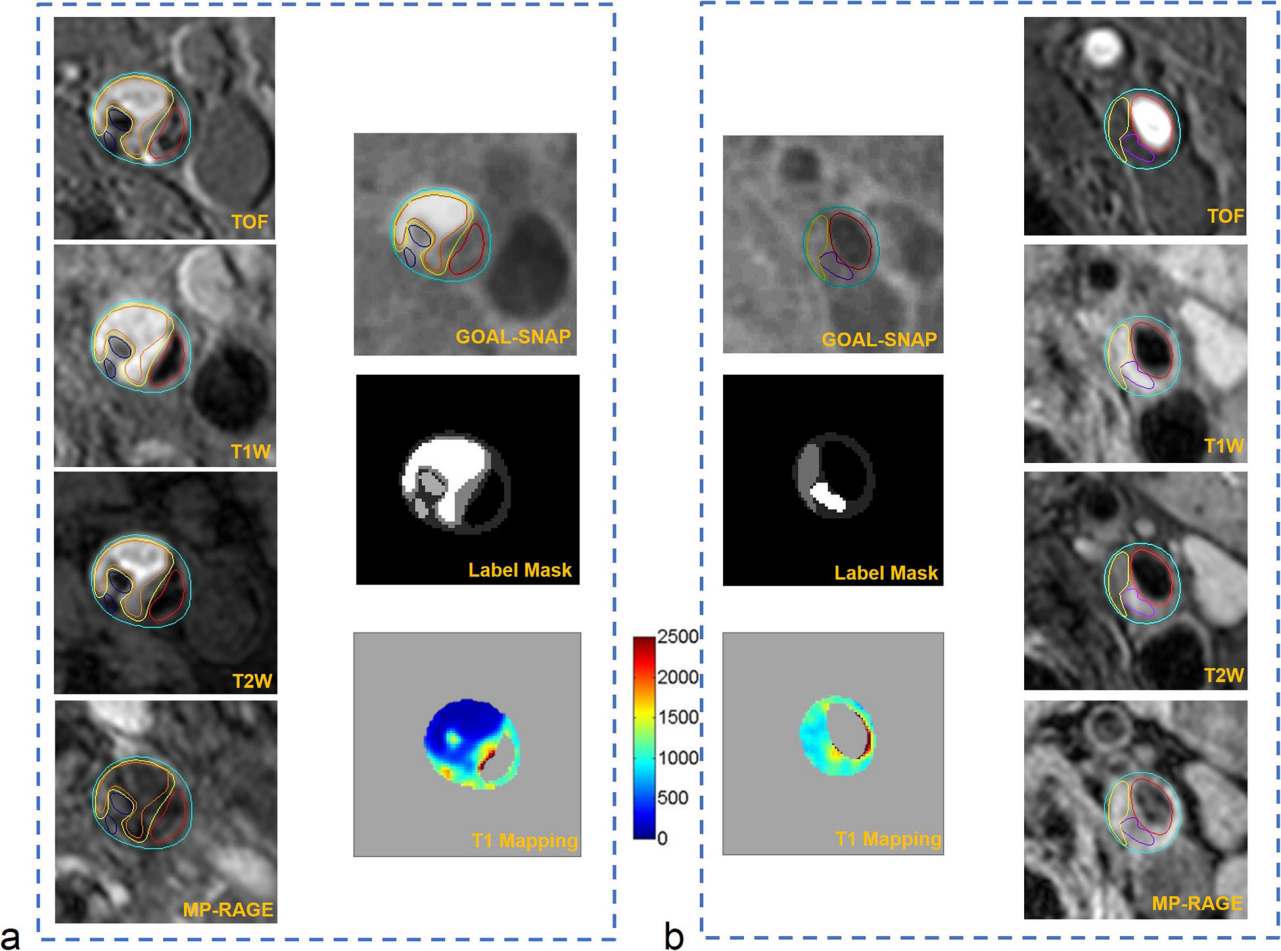
Multi-contrast CMR vessel wall images, SNAP with golden angle radial k-space sampling (GOAL-SNAP) images, label masks and T1 mappings of carotid plaques. The carotid plaques contained various components, including IPH (orange boundary), calcification (dark blue boundary), NC (yellow boundary) and LM (purple boundary). Intraplaque hemorrhage showed a reduced T1 compared with fibrous tissue (**a**). The T1 value of NC was shorter than that of LM (**b**)

In 34 patients who completed CMR imaging before CEA surgery, 818 slices from 66 carotid arteries with acceptable image quality (2 carotid arteries were excluded due to poor image quality) were analyzed. Of 66 carotid plaques from 34 patients, 19 (28.8%) had NC without IPH, 29 (43.9%) had IPH, and 36 (54.5%) had LM on CMR imaging. Of 818 analyzed slices, 285 (34.8%) had NC without IPH, 161 (19.7%) had IPH, and 166 (20.3%) had LM. The T1 values for LM, NC and IPH for each slice are detailed in Fig. 2a. Among all plaque components, IPH showed the shortest T1 value (907 ± 354 ms) and LM had the longest T1 value (1379 ± 340 ms) (all *p* ≤ 0.001). Typical carotid atherosclerotic plaque images of multi-contrast vessel wall imaging and T1 mapping are shown in Fig. 3.

### T1 values of plaque components validated by histology

**Fig. 4:**
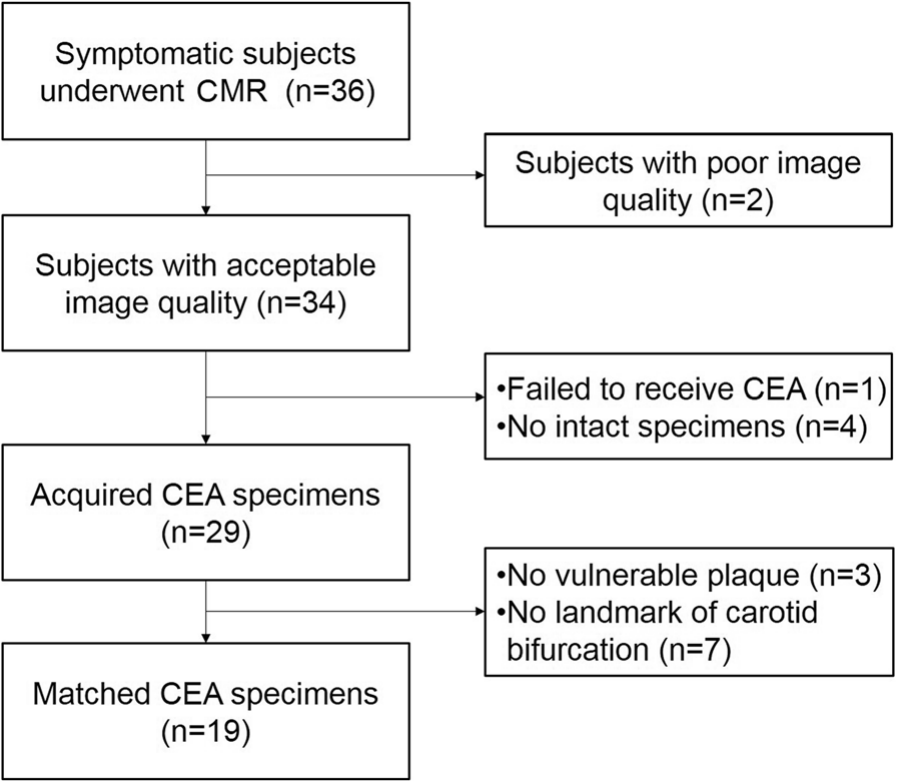
The detailed information about the recruitment of patients and carotid endarterectomy (CEA) specimens

During matching the histological sections of carotid plaque specimens with multicontrast CMR images, 3 specimens were found to have no complicated plaque compositions such as NC and IPH and 7 specimens didn’t have the landmark of carotid bifurcation. Therefore, 19 specimens were successfully used for histological validation of T1 mapping. The recruitment of patients and CEA specimens are detailed in Fig. 4.

In total, 52 slices from 19 CEA specimens of 19 subjects (mean age, 66.2 ± 7.1 years; 14 males) were included in the comparison study between histology and T1 mapping. The number of slices of each histological specimen was provided in Table 3. Of the 19 volunteers with histology data, 12 (63.2%) had NC, 14 (73.7%) had IPH, and 14 (73.7%) had LM. The mean T1 values of IPH, NC and LM for each subject were 685 ± 226 ms, 1171 ± 164 ms, and 1382 ± 301 ms, respectively. Of 52 slices, 25 (48.1%) had LM, 36 (69.2%) had IPH, and 24 (46.2%) had NC on histology. The mean T1 values of IPH, NC, and LM for each slice were 651 ± 253 ms, 1161 ± 182 ms, and 1447 ± 310 ms, respectively (all *p* ≤ 0.001, Fig. 2b). Of 36 slices with IPH, 14 (38.9%) and 22 (61.1%) were classified into Type 1 and Type 2 IPH, respectively. The T1 values for Type 1 and Type 2 IPH were 456 ± 193 ms and 775 ± 205 ms, respectively (*p* < 0.001). Of the 16 slices for the reproducibility study, 8 (50.0%) had NC, 12 (66.7%) had IPH, and 6 (37.5%) had LM. Of the 12 slices with IPH, 5 (41.7%) had Type 1 IPH and 7 (58.3%) had Type 2 IPH. The identification rate of IPH, NC and LM between two rounds of review by the two pathologists was same. There were excellent agreements of T1 value measurements (ICC = 0.972, 95% CI 0.937–0.987) and the classification of IPH (ICC = 0.911, 95% CI 0.689–0.974) between two rounds of review for the histological specimens.

**Table 3:** The number of slices of each histological specimen

Specimen ID	Number of slices	Specimen ID	Number of slices
1	4	11	4
2	2	12	3
3	4	13	2
4	1	14	3
5	5	15	1
6	4	16	4
7	3	17	2
8	1	18	3
9	2	19	2
10	2		

**Fig. 5:**
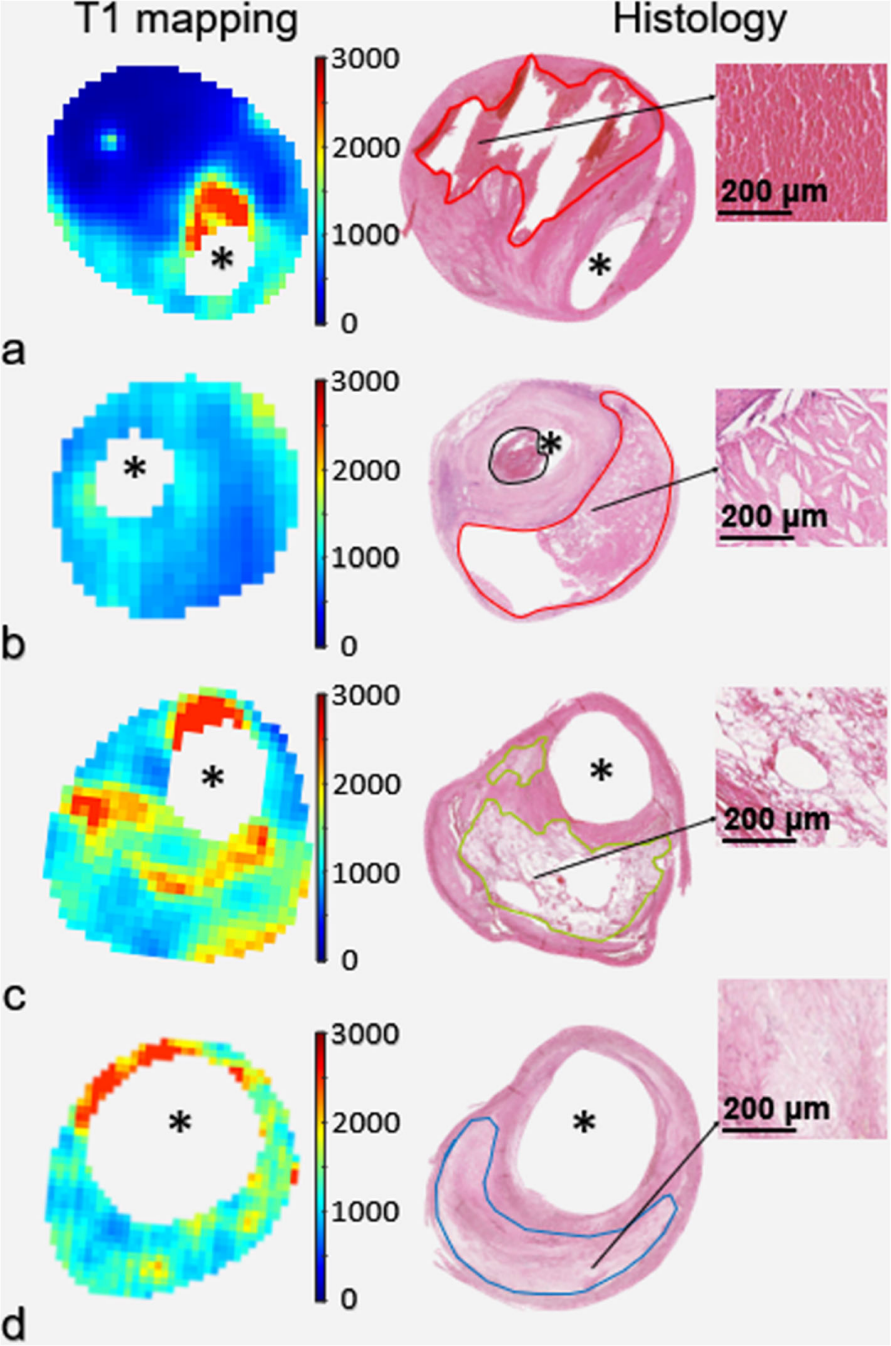
T1 mapping and H&E stained histology of carotid vulnerable plaques. Carotid vulnerable plaques with Type 1 intraplaque hemorrhage (red boundary, **a**), Type 2 IPH (red boundary, **b**), LM (green boundary, **c**), and NC (blue boundary, **d**) are shown, respectively. The black boundary was the mural thrombus inside the lumen

Figure 5 represents typical carotid plaque images with LM, IPH and NC on both histologic sections and T1 fitting images.

### Assessment of IPHs, LM and NC by T1 mapping validated by histology

**Fig. 6:**
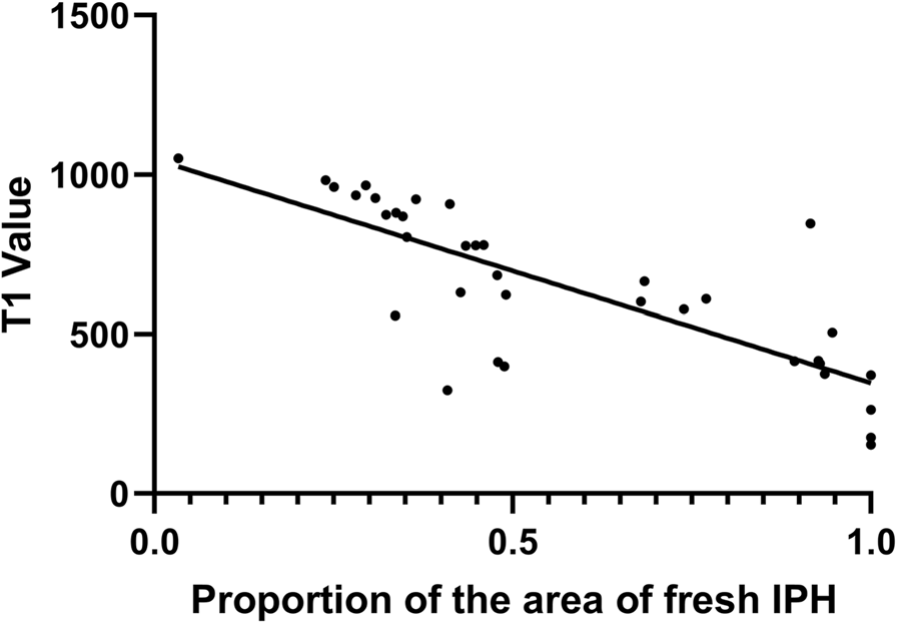
The regression line between T1 value and the proportion of the area of fresh hemorrhage

**Fig. 7:**
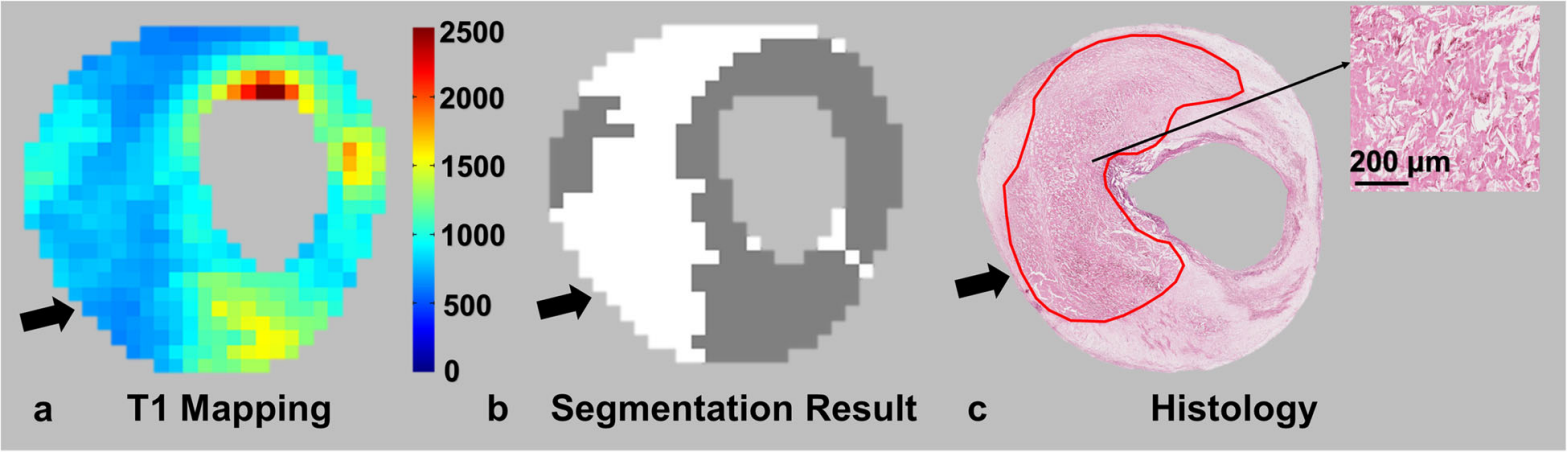
Typical images for the segmentation of IPHs (arrows). The areas of IPHs on T1 mapping and histology were 17.8 mm^2^ and 16.0 mm^2^, respectively

According to the T1 values of plaque components validated by histology on each slice, the cut-off values for the identification of IPH, NC and LM were set as ≤ 651 ms (mean T1 value of IPH), 1143–1180 ms (mean T1 value ± 1/10 standard deviation of NC) and 1416–1478 ms (mean T1 value ± 1/10 standard deviation of LM), respectively. The cut-off values for the classification of Type 1 IPH and Type 2 IPH were set as < 456 ms and 456–651 ms, respectively. Moderate agreement was found between T1 mapping and histology in identifying the presence of IPHs (kappa = 0.51, *p* < 0.001) and classifying the type of IPHs (kappa = 0.40, *p* = 0.028). The accuracy of T1 mapping in identifying and classifying IPHs was 78.8% and 70.0%, respectively (Table 4). The sensitivity and specificity of T1 mapping in identifying IPHs was 83.3% and 68.8%, respectively. Strong correlation was found between T1 values and the proportion of the area of fresh hemorrhage occupying the total area of IPH (*r* = 0.780, *p* < 0.001, Fig. 6). Excellent agreement between T1 mapping and histology can be observed in measuring the area of IPHs (ICC = 0.816, 95% CI 0.679–0.894). For IPHs detected by both T1 mapping and histological sections, the area of IPHs measured on T1 mapping was larger than that measured on histology, but no significant difference was found (13.7 ± 8.2 mm^2^ vs. 11.4 ± 7.2 mm^2^, *p* = 0.080). The typical images for the segmentation of IPH are shown in Fig. 7. With the reference of histology, the accuracy, sensitivity and specificity of T1 mapping were 53.8%, 60.0% and 48.1% in identifying LM, and 59.6%, 79.2% and 42.9% in identifying NC, respectively.

**Table 4:** The agreement between T1 mapping and histology in assessing IPHs

		Histology	
		Absence	Presence
T1 Mapping	Absence	11	6
	*Presence	5	30
		Type 1	Type 2
	Type 1	10	5
	Type 2	4	11

*Presence of IPH on T1 mapping was determined when the shortest T1 (the mean T1 value of four adjacent pixels) of the axial slice was shorter than the threshold. IPH: intraplaque hemorrhage

## Discussion

In this study, we calculated the T1 values of carotid atherosclerotic plaque components which were identified on multi-contrast vessel wall imaging or validated by histology. We found that T1 values of IPH, NC and LM were significantly different which suggests the potential capability of T1 mapping in the identification and segmentation of carotid plaque components. Moderate to good agreement was found in the identification, classification and quantitative measurements of IPHs between T1 mapping and histology.

In this study, significant difference in T1 values was found among NC, IPH and LM validated by multi-contrast CMR images or histological specimens. Few previous studies investigated the in-vivo T1 values of carotid plaque components due to the challenges of high-resolution vessel wall T1 mapping. Previously, ex-vivo intracranial plaques from 20 cadavers were studied in which the T1 values of NC, fibrous tissue, fibrous cap, wall and calcification were reported [8]. However, the ex-vivo plaques have different conditions, such as temperature and exchanges of molecules, compared with in-vivo ones. Recently, Ota et al. measured the longitudinal relaxation rates (R1 = 1/T1) of in-vivo carotid plaque components on pixel basis validated by histology [9]. The R1 of NC, IPH and fibrous tissue was reported to be 0.4–1.2 s^− 1^ (T1: 833–2500 ms), ≥ 1.5 s^− 1^ (T1 ≤ 667 ms), and 0.2–0.8 s^− 1^ (T1: 1250–5000 ms), respectively. In contrast, other in-vivo studies at 3 T reported that the T1 value for the vessel wall, muscle, and IPH was 1195 to 1213 ms, 1027 to 1219 ms, and 371 ± 93 ms (the minimum value), respectively [10, 18, 19]. The T1 value of NC in the present study was comparable with previous studies. In addition, the T1 value of IPHs which were identified on multi-contrast CMR images and histology was 907 ± 354 ms and 651 ± 253 ms, respectively. This difference might be caused by a larger proportion of old IPH which showed a longer T1 value than fresh IPH in multi-contrast imaging samples than that in histological specimens (66.1%). The difference might be contributed by the mismatched resolution between CMR imaging and histology. The IPHs identified on multi-contrast images might include part of NC which would cause a longer T1 value. The reported T1 values of LM or NC identified by multi-contrast imaging and histology were similar. Additional value of the present study was that the in-vivo T1 value of LM on 3 T validated by histology was firstly reported. The differentiable T1 values of carotid plaque components suggest the potential of automatic segmentation of plaque components using T1 mapping.

T1 mapping was found to have a moderate agreement with histology in identification and classification of carotid IPHs. In the present study, the IPHs were divided into two types according to the percentage of fresh hemorrhage on histology. We found that Type 1 IPH showed significantly shorter T1 value than Type 2 IPH and T1 values had strong correlation with the proportion of area of fresh hemorrhage occupying the total area of IPH, indicating that lower T1 values signify fresher hemorrhage which represents a higher risk of carotid plaque. Histologically, fresh IPH was mainly consisted of intact red blood cells, polymorphonuclear cells, lymphocytes, and scattered macrophages [7]. The intact red blood cell with intracellular methemoglobin in fresh IPH might shorten the T1 values. In addition, a threshold segmentation was also utilized to quantify carotid IPH and good agreement was found in measuring the area of IPHs between T1 mapping and histology in the present study. The threshold segmentation is appealing because it can be used across different centers. In the present study, T1 mapping images had a low accuracy and specificity in identification of NC and LM which might be caused by the overlaps of their T1 values. The NC has complex components including cholesterol crystal, debris of apoptotic cells and particles of calcium which are attributed to wide range of T1 value. LM shows a long T1 value which might be underestimated by the GOAL-SNAP imaging due to the intrinsic limitation of this technique for the long T1 value validated by spin-echo sequence [10]. Therefore, it was challenging to accurately identify and segment the NC and LM using T1 mapping. In addition, the identification and segmentation of plaque components using T1 mapping were based on the boundaries of arterial wall which dominantly determined by T1-QIR images of multi-contrast vessel wall imaging. Because the wall boundaries on T1 mapping images were not clear, the T1 mapping images need to be combined with at least one black blood T1 weighted sequence to identify plaque components.

There are several limitations in this study. First, the registration of CMR images and histology might be influenced by the mismatched resolution. The histological sections were excised every 0.5 mm with 10 μm thickness. In contrast, the thickness of CMR images was 2 mm. High-resolution CMR vessel wall imaging along with decreased partial volume effects is warranted in future studies. Second, fibrous cap, as a key feature of vulnerable plaques, was not evaluated due to the limited spatial resolution. A previous histological study showed that the minimum thickness of thin fibrous cap was 200 μm [20], suggesting that a higher resolution CMR imaging will be required for assessment of fibrous cap. Third, calcification was not evaluated in this study due to the low signal-to-noise ratio of calcification and reliable T1 cannot be fitted. Qi et al. set the value of T1 and T2 of calcification as zero to realize the segmentation of calcification [18]. Fourth, fibrous tissue was not evaluated in this study. Masson Trichrome stain for the assessment of fibrous tissue is warranted in the future. Fifth, the sample size of our study was relatively small and most of results were from slice-level analysis which might cause over/under representation of some subjects and introduce bias. A larger sample size with the consideration of inter-subject variability was warranted for the validation of T1 mapping in identifying plaque components with enough training and test datasets. Finally, histological sections were ex-vivo samples in which plaque components might have morphological changes to some extent. Gamble et al. reported that about 7.8% of shrinkage was determined in whole ex-vivo specimens [17]. However, the shrinkage of each plaque component has not been investigated. Furthermore, the ex-vivo specimens might have different shape compared with the in-vivo sections.

## Conclusions

CMR T1 values of carotid plaque components, particularly for IPH, are differentiable, and the stage of IPH can be classified according to T1 values, suggesting the potential capability of assessment of vulnerable plaque components by T1 mapping.

## Data Availability

The data that support the findings of this study are available on request from the corresponding author [Z.X.]. The data are not publicly available due to them containing information that could compromise research participant privacy/consent.

## Abbreviations

CEA: Carotid endarterectomy
CMR: Cardiovascular magnetic resonance
GOAL-SNAP: SNAP with golden angle radial k-space sampling sequence
IPH: Intraplaque hemorrhage
IR: Inversion recovery
KWIC: K-space weighted image contrast
LM: Loose matrix
MDIR: Multislice double inversion recovery
MP-RAGE: Magnetization prepared rapid gradient echo acquisition
NC: Necrotic core
QIR: Quadruple inversion recovery
SNAP: Simultaneous non-contrast angiography and intraplaque hemorrhage
T1w: T1 weighted
T2w: T2 weighted
TOF: Time of flight
